# Evaluation of Dental Pulp Stem Cell Heterogeneity and Behaviour in 3D Type I Collagen Gels

**DOI:** 10.1155/2020/3034727

**Published:** 2020-09-10

**Authors:** Amr Alraies, Rachel J. Waddington, Alastair J. Sloan, Ryan Moseley

**Affiliations:** ^1^Regenerative Biology Group, Oral and Biomedical Sciences, School of Dentistry, Cardiff Institute of Tissue Engineering and Repair (CITER), College of Biomedical and Life Sciences, Cardiff University, Cardiff, UK; ^2^Melbourne Dental School, Faculty of Medicine, Dentistry and Health Sciences, The University of Melbourne, Melbourne, Australia

## Abstract

Dental pulp stem cells (DPSCs) are increasingly being advocated for regenerative medicine-based therapies. However, significant heterogeneity in the genotypic/phenotypic properties of DPSC subpopulations exist, influencing their therapeutic potentials. As most studies have established DPSC heterogeneity using 2D culture approaches, we investigated whether heterogeneous DPSC proliferative and contraction/remodelling capabilities were further evident within 3D type I collagen gels *in vitro*. DPSC subpopulations were isolated from human third molars and identified as high/low proliferative and multipotent/unipotent, following *in vitro* culture expansion and population doubling (PD) analysis. High proliferative/multipotent DPSCs, such as A3 (30 PDs and 80 PDs), and low proliferative/unipotent DPSCs, such as A1 (17 PDs), were cultured in collagen gels for 12 days, either attached or detached from the surrounding culture plastic. Collagen architecture and high proliferative/multipotent DPSC morphologies were visualised by Scanning Electron Microscopy and FITC-phalloidin/Fluorescence Microscopy. DPSC proliferation (cell counts), contraction (% diameter reductions), and remodelling (MMP-2/MMP-9 gelatin zymography) of collagen gels were also evaluated. Unexpectedly, no proliferation differences existed between DPSCs, A3 (30 PDs) and A1 (17 PDs), although A3 (80 PDs) responses were significantly reduced. Despite rapid detached collagen gel contraction with A3 (30 PDs), similar contraction rates were determined with A1 (17 PDs), although A3 (80 PDs) contraction was significantly impaired. Gel contraction correlated to distinct gelatinase profiles. A3 (30 PDs) possessed superior MMP-9 and comparable MMP-2 activities to A1 (17 PDs), whereas A3 (80 PDs) had significantly reduced MMP-2/MMP-9. High proliferative/multipotent DPSCs, A3 (30 PDs), further exhibited fibroblast-like morphologies becoming polygonal within attached gels, whilst losing cytoskeletal organization and fibroblastic morphologies in detached gels. This study demonstrates that heterogeneity exists in the gel contraction and MMP expression/activity capabilities of DPSCs, potentially reflecting differences in their abilities to degrade biomaterial scaffolds and regulate cellular functions in 3D environments and their regenerative properties overall. Thus, such findings enhance our understanding of the molecular and phenotypic characteristics associated with high proliferative/multipotent DPSCs.

## 1. Introduction

Adult human dental pulp stem cells (DPSCs) are increasingly being characterised and evaluated as a viable mesenchymal stem cell (MSC) source in the development of effective regenerative medicine-based therapies for clinical use [1–3]. Such conclusions are based on DPSCs being readily accessible from the permanent dentition, in addition to their self-renewal, clonogenicity, and multilineage (e.g., osteogenic, chondrogenic, adipogenic, myogenic, and neurogenic) differentiation capabilities, comparable to those established for bone marrow-derived MSCs [4–6]. Consequently, DPSCs have already been shown to be beneficial to repair following transplantation into various animal model defects *in vivo*, related to diseases and traumas within clinical fields such as Dentistry, Orthopaedics, Neurology, Ophthalmology, and Cardiology [1–3].

In order to replenish MSC populations and facilitate *de novo* tissue repair, exogenously sourced MSCs are invariably transplanted into wound sites seeded within three-dimensional (3D) biomaterial scaffolds or hydrogels, which act as cell carriers and provide mechanical support. Furthermore, the plethora of synthetic, semisynthetic, and naturally derived scaffold materials currently used endeavor to recapitulate the physiochemical properties of the extracellular matrix (ECM) comprising the native stem cell niche microenvironment, which actively controls MSC self-renewal, proliferative, migratory, and differentiation responses [7–10]. Scaffold-based 3D assemblies provide a spatial arrangement that permit multiple cellular interactions with MSCs via integrin-based focal adhesions and Rho GTPases, which regulate cytoskeletal organization and cellular morphology, in addition to mediating cell signalling, mechanical force transduction, and adhesion to the biomaterial substratum [11, 12]. Most natural and semisynthetic biomaterial scaffolds are designed to undergo gradual remodelling and degradation by matrix metalloproteinases (MMPs), such as gelatinases MMP-2 and MMP-9, to be replaced by the newly synthesized tissue whilst still providing a functional role in supporting MSC activity [13–16].

Despite such advances, fundamental challenges still remain with the development of DPSC-based therapies for clinical applications, due to the significant heterogeneity between DPSCs isolated from dental pulp tissues, with individual subpopulations demonstrating major differences in proliferation and differentiation capabilities [4, 5, 17, 18]. Consequently, despite heterogeneous DPSC populations achieving >120 population doublings (PDs) *in vitro*, only 20% of purified DPSCs are capable of proliferating >20 PDs. Such features have significant implications for DPSC exploitation, as a major limitation of MSC-based therapies is the extensive *in vitro* expansion required to produce sufficient cell numbers for clinical use, which eventually leads to proliferative decline, replicative senescence, and impaired MSC regenerative properties [19, 20].

Recent studies by ourselves have demonstrated that whilst high proliferative DPSCs are capable of >80 PDs in culture, low proliferating DPSCs only completed <40 PDs before senescence, correlating with DPSCs with high proliferative capacities possessing longer telomeres than less proliferative subpopulations [21, 22]. Low proliferative DPSC senescence was also associated with the loss of stem cell marker characteristics and impaired osteogenic/chondrogenic differentiation, favouring adipogenesis. In contrast, high proliferative DPSCs retained multipotency capabilities, only demonstrating impaired differentiation following prolonged *in vitro* expansion (>60 PDs).

Therefore, due to the established genotypic and phenotypic heterogeneity previously demonstrated to exist between high proliferative/multipotent and low proliferative/unipotent DPSC subpopulations utilising 2D monolayer cultures, this study investigated whether similar DPSC heterogeneity existed in terms of their respective proliferation and abilities to remodel/contract 3D type I collagen gels *in vitro*. Specifically, the proliferative and ECM contraction/remodelling capabilities of a high proliferative/multipotent DPSC subpopulation, A3, were evaluated at early (nonsenescent) stages (at 30 PDs) and later (approaching senescence) stages (at 80 PDs) in its proliferative lifespan and compared against a low proliferative/unipotent DPSC, A1, approaching senescence (at 17 PDs). Type I collagen gels were employed, as they are extensively used in the development of natural biomaterial scaffolds for regenerative medicine applications [23, 24].

## 2. Materials and Methods

### 2.1. Dental Pulp Stem Cell Isolation and Characterisation of Proliferation/Differentiation Capabilities in 2D Monolayer Culture

Human DPSCs were isolated from third molar teeth collected from patients (all female, age 18-30 years) undergoing orthodontic extractions at the School of Dentistry, Cardiff University, UK. Teeth were collected in accordance with the Declaration of Helsinki (2013), with informed patient consent and ethical approval by the South East Wales Research Ethics Committee of the National Research Ethics Service (NRES), UK.

Single cell suspensions of dental pulp tissues were obtained, with DPSCs preferentially selected and isolated from cell suspensions by differential fibronectin adhesion assay [21]. Isolated cells were confirmed as DPSCs through cell surface marker expression (positive for MSC markers, CD73, CD90, and CD105, negative for hematopoietic stem cell marker, CD45). DPSCs subsequently underwent extended culture expansion and characterisation as being high or low proliferative and multi- or unipotent. Proliferation analysis was based on the PDs reached by each DPSC subpopulation prior to senescence, confirmed by the detection of other senescence-related markers, including reduced telomere lengths, positive senescence-associated *β*-galactosidase staining, and increased p53, p21^waf1^, and p16^INK4a^ expression [21].

Differentiation analyses were based on the abilities of each DPSC subpopulation to undergo osteogenic, chondrogenic, and adipogenic differentiation, via detection of established differentiation markers as previously described [21]. Individual DPSC subpopulations were subsequently confirmed as being high proliferative/multipotent DPSCs (such as A3, capable of >80 PDs) or low proliferative/unipotent DPSCs (such as A1, capable of <40 PDs).

### 2.2. Preparation and Culture of 3D Type I Collagen Gels

DPSCs were seeded in type I collagen gels, based on the method of Stephens et al. [25]. Type I rat tail collagen (>2 mg/mL, First Link, Wolverhampton, UK) was reconstituted in an *α*-modified Minimum Essential Medium (*α*MEM), containing ribonucleosides and deoxyribonucleosides, supplemented with 4 mM L-glutamine, 100 U/mL penicillin G sodium, 0.1 *μ*g/mL streptomycin sulphate, 0.25 *μ*g/mL amphotericin (all ThermoFisher Scientific, Paisley, UK), and 100 *μ*M L-ascorbate 2-phosphate (Sigma, Poole, UK), to provide 1 mg/mL solutions. Collagen solutions were neutralised by the dropwise addition of 5 M sodium hydroxide. Subsequently, aliquots (1 mL) of the collagen solutions were added into each well of 12-well plate and maintained at 37°C/5% CO_2_ for 45 min. High proliferative/multipotent DPSC subpopulations, A3, at early (nonsenescent, 30 PDs) and late (approaching senescence, 80 PDs) stages in their proliferative lifespans, and low proliferative/unipotent DPSCs, A1 (approaching senescence, 17 PDs), were suspended in *α*MEM, containing 20% gelatinase-free, foetal calf serum (FCS, ThermoFisher Scientific, prepared from complete FCS according to [26]). DPSCs (5 × 10^4^ cells/mL) in gelatinase-free *α*MEM were subsequently added to the collagen gels. Collagen gels (*n* = 3 per DPSC subpopulation) were maintained at 37°C/5% CO_2_ for 12 days, with gels remaining either attached to the surrounding tissue culture plastic or detached at Day 0 using a sterile filter tip. Acellular type I collagen gels were also established as controls.

### 2.3. Scanning Electron Microscopy (SEM) Analysis of Acellular Collagen Gels

The architecture of type I collagen fibres within the acellular gels was assessed by Scanning Electron Microscopy (SEM). Acellular collagen gels were established as described above and fixed with 2.5% glutaraldehyde (Agar Scientific, Stansted, UK) in 0.1 M cacodylate buffer for 2 h at room temperature. Gels were subsequently washed (×3) with 0.1 M cacodylate buffer and dehydrated through a graded ethanol series: 30%, 50%, 70%, and 100% for 15 min each, and gels allowed to dry overnight. Dried hydrogels were sputter-coated and SEM images taken at multiple locations throughout each gel, using a S4800 Scanning Electron Microscope (Hitachi, Tokyo, Japan), operated at 10 kV.

### 2.4. DPSC Proliferation in Type I Collagen Gels

Collagen gels were also established as described above, for the high proliferative/multipotent DPSC subpopulation, A3, at early (non-senescent, 30 PDs) and late (approaching senescence, 80 PDs) stages in their proliferative lifespans, and low proliferative/unipotent DPSCs, A1 (approaching senescence, 17 PDs), in order to assess their respective proliferative capabilities over 12 days in culture. DPSCs were recovered at Days 1, 2, 4, 6, and 12 from collagen gels by enzymatic digestion, as previously described [25]. At each time point, collagen gels were solubilized with collagenase A (400 *μ*L, 2 mg/mL, Roche, West Sussex, UK), at 37°C/5% CO_2_ for 2 h. This was followed by incubation with 0.05% trypsin/0.53 mM EDTA (100 *μ*L, ThermoFisher Scientific), at 37°C/5% CO_2_ for 20 min. DPSCs were recovered by centrifugation (1,500 g/5 min) and viable cell numbers determined using a Neubauer haemocytometer, with 0.02% Trypan Blue solution (Sigma). Each experiment was performed on 3 separate occasions.

### 2.5. Type I Collagen Gel Contraction by DPSCs

DPSC-seeded collagen gels were established as described above. The relative extents of remodelling and contraction of the type I collagen gels by high proliferative/multipotent DPSC subpopulation, A3, at early (nonsenescent, 30 PDs) and late (approaching senescence, 80 PDs) stages in their proliferative lifespans, and low proliferative/unipotent DPSCs, A1 (approaching senescence, 17 PDs), were quantified from 3 separate gel diameter measurements performed on each replicate sample (*n* = 3 per DPSC subpopulation), at Days 1, 2, 4, 6, and 12 in the culture. Average contraction values obtained were expressed as % reduction in gel diameter, compared to gel diameters at 0 h. Conditioned medium was also collected from each individual well for the analysis of relative gelatinase (MMP-2 and MMP-9) activities at the same time points as the gel diameter measurements. Each experiment was performed on 3 separate occasions.

### 2.6. Assessment of Gelatinase (MMP-2 and MMP-9) Activities

The relative amounts of pro- and active MMP-2 and MMP-9 activities produced by high proliferative/multipotent DPSC subpopulations, A3, at early (nonsenescent, 30 PDs) and late (approaching senescence, 80 PDs) stages in their proliferative lifespans, and low proliferative/unipotent DPSCs, A1 (approaching senescence, 17 PDs), in type I collagen gels, were determined by gelatin zymography based on the method of Stephens et al. [27]. Collected conditioned media (25 *μ*L) were mixed with equal volumes of nonreducing Laemmli loading buffer and separated by SDS-polyacrylamide gel electrophoresis (SDS-PAGE), on precast 10% gelatin zymography gels (Ready Gel 10% Gelatin Zymogram Gels, Bio-Rad Laboratories, Hemel Hempstead, UK), using a Mini-PROTEAN® Tetra Cell System (Bio-Rad Laboratories) at 20 mA. SDS was removed from each SDS-PAGE gel by washing in 2.5% Triton X-100 solution (Sigma), for 1 h at room temperature. Following electrophoresis, gelatinases within the SDS-PAGE gels were activated by incubation in 25 mM Tris-HCl buffer, pH 7.6, containing 5 mM calcium chloride (Sigma), 25 mM sodium chloride (ThermoFisher Scientific), and 5% Brij 35 (Sigma), at 37°C overnight. Gels were stained with 0.05% Coomassie Blue solution (Sigma) in 12% acetic acid and 54% methanol (both ThermoFisher Scientific) and destained in 7.5% acetic acid/5% methanol solution. Gel images were captured, with MMP-2 and MMP-9 identified by the appearance of clear bands at comparable molecular weights to loaded MMP-2 and MMP-9 standards, respectively. The relative amounts of MMP-2 and MMP-9 in captured images were determined by densitometry, using ImageJ® Software (https://imagej.nih.gov/ij/). Each experiment was performed on 3 separate occasions.

### 2.7. SEM and Fluorescence Microscopy Analysis of DPSC Morphologies in Collagen Gels

The cellular morphologies of the nonsenescent (30 PDs), high proliferative/multipotent DPSC subpopulation, A3, in type I collagen gels, were examined by both SEM and via actin cytoskeleton staining using FITC-phalloidin and Fluorescence Microscopy. DPSC-seeded collagen gels were established, as described above. For SEM, collagen gels were fixed, processed, and visualised, as per the methods detailed above. For Fluorescence Microscopy, DPSC-seeded collagen gels were washed with phosphate-buffered saline (PBS, 1 × 1 mL) and fixed with 4% paraformaldehyde (1 mL, Santa Cruz, Dallas, USA) for 10 min at room temperature. Gels were washed with PBS (2 × 1 mL) at 5 min interval and treated with 0.3% Triton X-100 (1 mL) for 30 min at room temperature, under constant agitation. Gels were washed with Tris-buffered saline (TBS, pH 7.5, 2 × 1 mL) and blocked with 1% bovine serum albumin (BSA, Fraction V, ThermoFisher Scientific) in TBS (1% BSA-TBS) for 1 h at room temperature, under constant agitation. DPSC-seeded gels were stained with FITC-phalloidin (20 *μ*g/mL, 1 mL, Sigma) in 1% BSA-TBS and incubated for 1 h at 4°C, under darkness and constant agitation. Gels were washed with TBS (2 × 1 mL) at 5 min interval and images captured by Fluorescence Microscopy (Olympus Provis AX70 Microscope, Olympus UK Ltd., Southend-on-Sea, UK).

### 2.8. Statistical Analysis

Each experiment was performed on *n* = 3 independent occasions. Data were expressed as mean ± standard error of mean (SEM). Graphical data were initially confirmed as exhibiting homoscedasticity using Levene's test. Having shown that the averages for all experimental group data were considered equal, graphical data were subsequently compared statistically by Analysis of Variance (ANOVA), performed using GraphPad InStat 3 (GraphPad Software Inc., La Jolla, USA). Statistical significance was considered at *p* < 0.05.

## 3. Results

### 3.1. SEM Analysis of Acellular Collagen Gels

**Figure 1:**
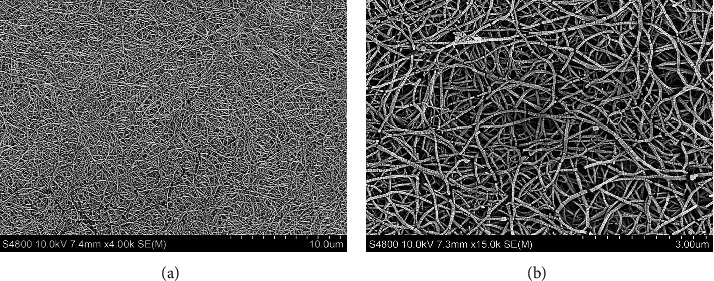
SEM images of the acellular type I collagen gels established using the protocols described herein, at (a) ×4,000 and (b) ×15,000 magnifications.

Representative SEM images of the acellular collagen gels established using the protocols described above are shown in Figures 1(a) and 1(b). The matrix architecture is typical for type I collagen gels of this nature, with the formation of a dense fibrillary network of narrow, elongated, and crosslinked collagen fibres [28, 29]. Consequently, the collagen fibre arrangements produced resulted in relatively small pore sizes being formed within the collagen gels overall.

### 3.2. DPSC Proliferation in Type I Collagen Gels

**Figure 2:**
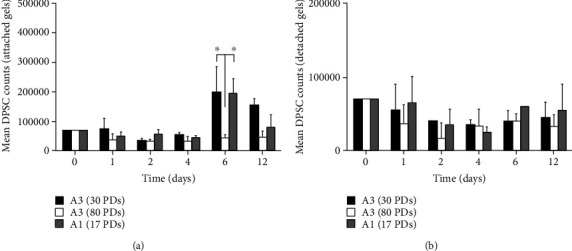
Mean cell numbers for high proliferative/multipotent DPSC subpopulation, A3, at early (nonsenescent, 30 PDs) and late (approaching senescence, 80 PDs) stages in their proliferative lifespans, and low proliferative/unipotent DPSCs, A1 (approaching senescence, 17 PDs), following seeding in (a) attached and (b) detached type I collagen gels over 12 days in culture. *N* = 3, mean ± SEM, ^∗^*p* < 0.05.

Mean cell numbers obtained for high proliferative/multipotent DPSC subpopulations, A3, at early (nonsenescent, 30 PDs) and late (approaching senescence, 80 PDs) stages in their proliferative lifespans, and low proliferative/unipotent DPSCs, A1 (approaching senescence, 17 PDs), in attached and detached type I collagen gels over 12 days in culture, are shown in Figure 2. For attached collagen gels, all DPSC subpopulations exhibited similar cell number profiles for the first 4 days (Figure 2(a)). However, A3 (30 PDs) and A1 (17 PDs) subsequently began to increase in cell number to Day 6, followed by reductions in cell numbers by Day 12 (all *p* > 0.05 between A3, 30 PDs, and A1, 17 PDs). In contrast, A3 (80 PDs) failed to exhibit any obvious increases in cell number beyond Day 4 (*p* < 0.05 versus A3, 30 PDs, and A1, 17 PDs, at Day 6).

With detached collagen gels, DPSCs A3 (30 PDs) and A1 (17 PDs) did not show any apparent increases in cell number during the 12 days in culture, unlike the scenario under attached type I collagen gel conditions (all *p* > 0.05, Figure 2(b)). However, A3 (80 PDs) demonstrated comparable cell number profiles under detached collagen gel conditions, to their attached gel counterparts over the 12-day culture period (all *p* > 0.05).

### 3.3. Type I Collagen Gel Contraction by DPSCs

**Figure 3:**
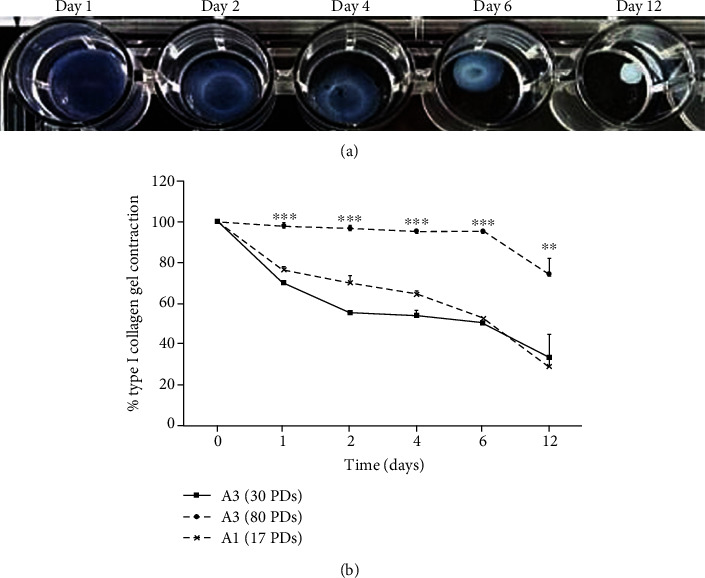
(a) Images of detached type I collagen gel contraction by nonsenescent (30 PDs), high proliferative/multipotent DPSC subpopulation, A3, over 12 days in culture. (b) Mean % detached type I collagen gel contraction by high proliferative/multipotent DPSC subpopulation, A3, at early (nonsenescent, 30 PDs) and late (approaching senescence, 80 PDs) stages in their proliferative lifespans, and low proliferative/unipotent DPSCs, A1 (approaching senescence, 17 PDs), over 12 days in culture. *N* = 3, mean ± SEM, ^∗∗∗^*p* < 0.001, ^∗∗^*p* < 0.01.

Representative type I collagen gel contraction images for nonsenescent (30 PDs), high proliferative/multipotent DPSC subpopulations, A3, over 12 days in culture are shown in Figure 3(a). Comparisons of mean collagen contraction values, expressed as the % reduction in gel diameter compared to Day 0, demonstrated that all DPSC subpopulations maintained in attached collagen gels over the 12-day culture period did not exhibit any gel contraction due to collagen gel attachment to the adjacent tissue culture plastic (all *p* > 0.05; *data not shown*). In contrast, DPSC subpopulations in detached type I collagen gels with high proliferative/multipotent DPSC subpopulations, A3, at early (nonsenescent, 30 PDs) and late (approaching senescence, 80 PDs) stages in their proliferative lifespans, and low proliferative/unipotent DPSCs, A1 (approaching senescence, 17 PDs), showed variable contraction rates over time (Figure 3(b)). For both A3 (30 PDs) and A1 (17 PDs), gels displayed gradual decreases in diameter/contraction over the initial 4 days in culture, although gels seeded with A3 (30 PDs) displayed greater contraction, compared to A1 (17 PDs). However, from Day 6 onwards, gels containing A1 (17 PDs) exhibited equivalent decreases in diameter/contraction to A3 (30 PDs) gels. Consequently, no significant differences were identified in the rates of type I collagen contraction between gels seeded with A3 (30 PDs) and A1 (17 PDs) throughout the 12-day culture period (all *p* > 0.05). In contrast to A3 (30 PDs) and A1 (17 PDs), gels seeded with A3 (80 PDs) displayed almost no shrinkage over the first 6 days in culture, with only a total reduction in diameter of ≅26% from its original size by Day 12 (Figure 3(b)). As such, gels containing DPSC subpopulation A3 (80 PDs) demonstrated significantly delayed gel contraction (all *p* < 0.001‐0.01), compared to those seeded with A3 (30 PDs) and A1 (17 PDs).

### 3.4. Gelatinase Activities in DPSC-Seeded Collagen Gels

**Figure 4:**
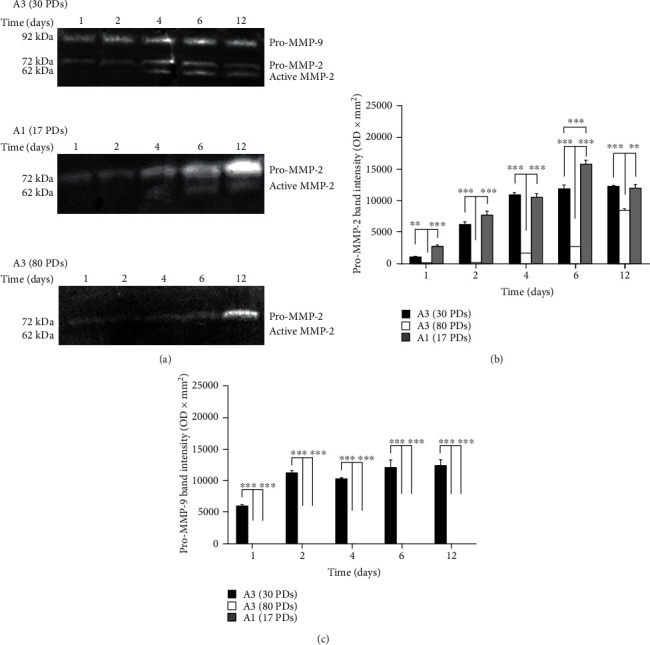
(a) Representative gelatin zymography gel images of pro- and active MMP-9/MMP-2 activities for high proliferative/multipotent DPSC subpopulation, A3, at early (nonsenescent, 30 PDs) and late (approaching senescence, 80 PDs) stages in their proliferative lifespans, and low proliferative/unipotent DPSCs, A1 (approaching senescence, 17 PDs), following seeding in detached type I collagen gels over 12 days in culture. Pro-MMP-2, active MMP-2, and pro-MMP-9 were detectable at 72 kDa, 62 kDa, and 92 kDa, respectively. Densitometry quantification of (b) pro-MMP-2 and (c) pro-MMP-9 activities for high proliferative/multipotent DPSC subpopulation, A3 (30 PDs and 80 PDs), and low proliferative/unipotent DPSC subpopulation (A1, 17 PDs), following seeding in detached type I collagen gels over 12 days in culture. *N* = 3, mean ± SEM, ^∗∗∗^*p* < 0.001, ^∗∗^*p* < 0.01.

Representative MMP-2 and MMP-9 gelatin zymography images for high proliferative/multipotent DPSC subpopulations, A3, at early (nonsenescent, 30 PDs) and late (approaching senescence, 80 PDs) stages in their proliferative lifespans, and low proliferative/unipotent DPSCs, A1 (approaching senescence, 17 PDs), in detached type I collagen gels over 12 days in culture, are shown in Figure 4. MMP zymography demonstrated that pro-MMP-2 was detectable for all DPSC subpopulations analysed, being detectable from Day 1 onwards, whilst active MMP-2 was only detectable with A3 (30 PDs) and A1 (17 PDs, Figure 4(a)). Although MMP-2 activities gradually increased over time in culture, A3 (80 PDs) demonstrated the lowest levels of detectable pro- and active MMP-2 overall (Figure 4(b)), being significantly less than A3 (30 PDs) and A1 (17 PDs) at all time points (*p* < 0.001‐0.01 for pro-MMP-2). However, significant differences in pro-MMP-2 activities between A3 (30 PDs) and A1 (17 PDs) were only determined at Day 6 (*p* < 0.001). In contrast to MMP-2, A3 (30 PDs) was the only DPSC subpopulation to exhibit detectable pro-MMP-9 activities, being significantly higher than A1 (17 PDs, all *p* < 0.001) and A3 (80 PDs, all *p* < 0.001) throughout the culture duration (Figures 4(a) and 4(c)).

### 3.5. DPSC Morphologies in Type I Collagen Gels

**Figure 5:**
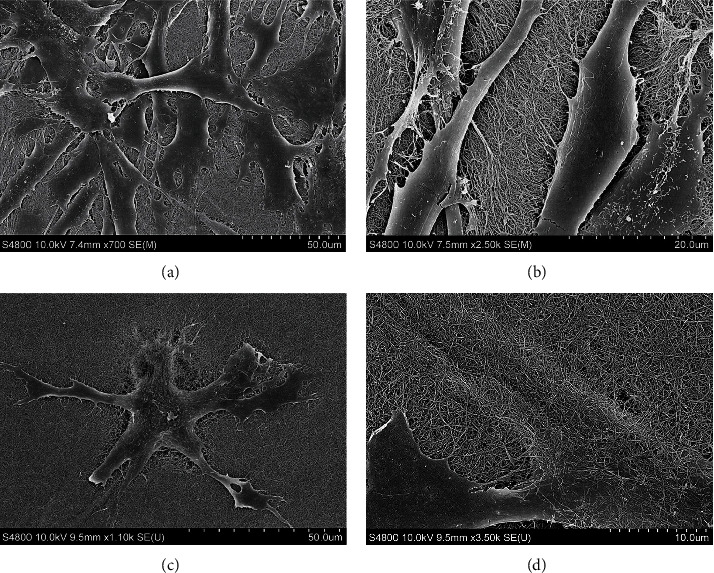
SEM images of nonsenescent (30 PDs), high proliferative/multipotent DPSC subpopulation, A3, following seeding in type I collagen gels at (a) Day 1 (×700 magnification), (b) Day 1 (×2,500 magnification), (c) Day 12 (×1,100 magnification), and (d) Day 12 (×3,500 magnification).

As a consequence of the rapid detached collagen gel contraction and unique MMP-9 expression/activity properties of the nonsenescent (30 PDs), high proliferative/multipotent DPSC subpopulation, A3, the cell morphologies of this subpopulation were further examined in type I collagen gels by SEM and Fluorescence Microscopy. SEM analysis demonstrated that DPSCs possessed good biocompatibility with the collagen gels overall, with viable cells detectable throughout the 12-day culture duration. DPSCs were shown to attach to collagen fibres and begin to spread at Day 1, with DPSCs largely displaying flattened, spindle-shaped, or polygonal morphologies overall (Figures 5(a) and 5(b)). At Day 12, DPSCs had spread further resulting in predominantly larger and more irregular 3D cuboidal or polygonal morphologies, due to superior interactions with the collagen matrices compared to Day 1, through numerous contacts between cell extensions and collagen fibres and the propagation of short pseudopodia-like protrusions into the ECM from the cell membranes (Figures 5(c) and 5(d)).

**Figure 6:**
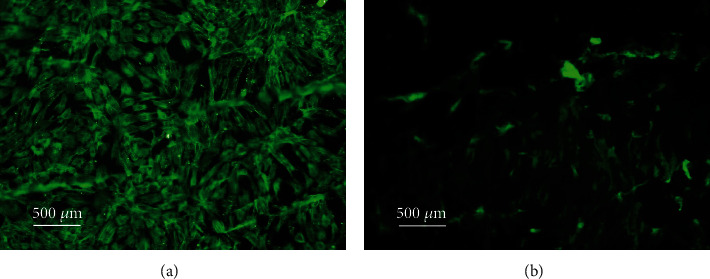
Fluorescence Microscopy images of FITC-phalloidin cytoskeletal staining of nonsenescent (30 PDs), high proliferative/multipotent DPSC subpopulation, A3, following seeding in (a) attached type collagen gels and (b) detached type collagen gels, at Day 1. ×100 magnification. Scale bar = 500 *μ*m.

Representative Fluorescence Microscopy images of high proliferative/multipotent DPSC subpopulation, A3 (30 PDs), cytoskeletal properties and morphologies in attached and detached type I collagen gels at Day 1 are shown in Figure 6. DPSCs exhibited limited morphological differences throughout culture overall. However, whereas DPSCs in attached type I collagen gels presented very clear actin stress fibres with a fibroblastic-like appearance (Figure 6(a)), DPSCs maintained in detached collagen gels appeared to lose their fibroblastic appearance with less prominent actin fibres (Figure 6(b)).

## 4. Discussion

This study aimed to determine whether the well-established variations in the proliferative, differentiation and other stem cell properties between different DPSC subpopulations in the 2D monolayer culture were further identifiable in 3D cultures within type I collagen gels. The concept of DPSC heterogeneity is well-established, with the presence of DPSC subpopulations in dental pulp tissues possessing contrasting proliferative and differentiation capabilities [4, 5, 17, 18]. Furthermore, recent corroborating findings have reported key differences between DPSCs in the relative susceptibilities to replicative senescence, correlating with contrasting telomere lengths and the differentiation capabilities of individual subpopulations [21, 22]. However, despite differences in the genotypic/phenotypic characteristics of individual DPSC subpopulations *in vitro*, to date, no studies had directly confirmed whether similar differences in responses exist when such DPSCs are seeded within 3D type I collagen gels. Therefore, the present study compared the proliferative and ECM contraction/remodelling capabilities of high proliferative/multipotent DPSC subpopulations, such as A3, at early (nonsenescent) stages (at 30 PDs) and later (approaching senescence) stages (at 80 PDs) in its proliferative lifespan, versus low proliferative/unipotent DPSCs, such as A1, whilst approaching senescence (at 17 PDs).

A great deal of evidence exists confirming that cells respond differently when cultured within 3D gel constructs, in comparison to the 2D monolayer culture, ascribed to the similarities between the 3D gel environment and the natural ECM comprising the MSC niche [8]. Furthermore, the status of 3D type I collagen and other gel types, in terms of whether these are detached or remain attached to the surrounding tissue culture plastic, has been previously shown to regulate cellular behaviour, including morphology, proliferation, and differentiation, in addition to contraction itself [30–34]. SEM and Fluorescence Microscopy analyses demonstrated that DPSCs, such as the high proliferative/multipotent subpopulation, A3, at nonsenescent stages in its proliferative lifespan (at 30 PDs), readily interacted and spread within type I collagen gels, particularly possessing spindle-shaped, fibroblast-like morphologies with obvious actin stress fibres, during early culture in attached type I collagen gels. More polygonal DPSC morphologies developed during prolonged culture due to superior interactions with the type I collagen matrices. In contrast, this DPSC subpopulation lost its fibroblast-like morphologies with less prominent actin fibres evident, when seeded within detached type I collagen gels. Collagen gel contraction by resident cells causes mechanical forces to be induced within the surrounding ECM, capable of influencing cellular alignment depending on the forces encountered [30]. As described below, type I collagen gel contraction by this DPSC subpopulation was particularly apparent during early culture stages, such as between Day 0 and Day 1. Thus, it was hypothesized that any prominent differences in the cytoskeletal organization between the attached and detached type I collagen gels would be particularly evident by Day 1, as was proven to be the case. Hence, as evident here, cells within attached gels alone develop isometric tension by forming prominent cytoskeletal actin stress fibres and focal adhesions with the surrounding ECM, permitting pseudopodia to aid their attachment or movement within the type I collagen environment [35–37].

Various cell types have also previously been shown to undergo mechanical stress-induced proliferation within attached collagen gels [31–33]. Despite increased DPSC numbers towards the latter stages of culture within attached type I collagen gels, relatively modest increases in DPSC proliferation were evident overall throughout the 12-day culture period. In contrast, detached gels showed minimal proliferation for all DPSC subpopulations analysed [38, 39]. However, cell number comparisons between high proliferative/multipotent DPSC subpopulations, A3, at early (nonsenescent, 30 PDs) and late (approaching senescence, 80 PDs) stages in their proliferative lifespans, and low proliferative/unipotent DPSCs, A1 (approaching senescence, 17 PDs), within attached type I collagen gels, revealed significantly impaired A3 (80 PDs) proliferative responses in 3D culture, compared to earlier in their proliferative lifespan (30 PDs). Intriguingly, no significant differences in proliferation were identifiable between A3 (30 PDs) and A1 (17 PDs), despite considerable differences in their proliferative capacities and susceptibilities to replicative senescence being previously identified in the 2D monolayer culture (>80 PDs versus <40 PDs, respectively; [21]). Therefore, obvious differences in proliferative capacities between A3 (80 PDs) and A3 (30 PDs) may be accounted for by the relative stages in their proliferative lifespan where this DPSC subpopulation was assessed, although the precise reasons behind such discrepancies in the relative proliferative responses of A3 (30 PDs) and A1 (17 PDs) between 2D and 3D environments remain to be elucidated.

Although studies have determined that cells proliferate poorly in detached gels due to their growth arrest within the G_0_/G_1_ phase of the cell cycle [40], the principle reasons underlying the maintenance of cellular proliferative capabilities within attached gels remain to be fully determined. Nonetheless, cell signalling pathways, such as ERK or MAP kinases, are acknowledged to respond to various stimuli, including mechanical stress, whilst such signalling cascades are not initiated in detached collagen gels, due to the absence of isometric tension and cytoskeletal actin stress fibre formation [41, 42]. Therefore, it is plausible that similar differences in ERK and MAPK signalling exist between the DPSC subpopulations within attached and detached type I collagen gels herein and warrant further investigation.

Cellular functions within the stem cell niche or biomaterial scaffolds are tightly regulated by the ECM microenvironment, remodelled by proteinases such as MMPs [43, 44]. Of the different MMP groups known, gelatinases MMP-2 and MMP-9 have common roles in mediating MSC remodelling of the niche and biomaterial scaffold degradation, thereby facilitating cellular activities [13–16]. Analysis of the relative abilities of high proliferative/multipotent DPSC subpopulations, A3, at early (nonsenescent, 30 PDs) and late (approaching senescence, 80 PDs) stages in their proliferative lifespans, and low proliferative/unipotent DPSCs, A1 (approaching senescence, 17 PDs), to remodel and contract detached type I collagen gels, demonstrated initial rapid collagen gel contraction with A3 (30 PDs), although comparable rates of gel contraction between A3 (30 PDs) and A1 (17 PDs) were identified overall during the 12-day culture duration. In contrast, A3 (80 PDs) displayed significantly impaired gel contraction capabilities, as a likely consequence of this being at the latter stages in its proliferative lifespan and more senescent than its lesser senescent A3 (30 PDs) counterparts. Such gel contraction findings were ascribed to contrasting MMP-2 and MMP-9 activities detected for each DPSC subpopulation, with significantly elevated levels of pro- and active forms of MMP-2 and MMP-9 being responsible for collagen gel contraction by a high proliferative/multipotent DPSC subpopulation, A3 (30 PDs), and pro- and active forms of MMP-2 alone for gel contraction by a low proliferative/unipotent DPSC subpopulation, A1 (17 PDs). However, A3 (80 PDs) demonstrated significant reduced pro- and active MMP-2 and MMP-9 activities overall.

Similarly impaired type I collagen gel reorganizational capabilities have previously been reported with other senescent cell populations, partly as a consequence of reduced MMP-2 expression and activities [45, 46]. Replicative senescence is also well-established to induce significant alterations in MSC genotype and phenotype, leading to impaired cellular regenerative properties and signalling mechanisms via the secretome associated with the senescence-associated secretory phenotype (SASP) [20, 47]. However, despite the SASP being accompanied by excessive expression of proinflammatory mediators, including MMPs, excessive MMP-2 and/or MMP-9 activities by more senescent A3 (80 PDs) were not detectable herein, as DPSC subpopulation, A3, lost its abilities to express MMP-2 and MMP-9 with extensive culture expansion in line with previous findings [45, 46]. Furthermore, in addition to limited type I collagen gel contraction and MMP-2/MMP-9 activities by A3 (80 PDs), superior remodelling efficiencies were only evident with A3 (30 PDs) at certain time points, compared to the more senescent subpopulation, A1 (17 PDs).

Although there are many similarities between gelatinases, MMP-2 and MMP-9, in terms of their structures and substrates, differences in their cellular sources, regulation, and activation do exist [43, 44]. Gelatinase expression and activation are regulated by numerous factors, including cytokines and growth factors. Mechanical stress is also a key factor capable of regulating MMP expression and activities within 3D gel environments during contraction, whilst MSC functions have also been shown to be highly regulated by MMPs [14, 16, 48–51]. Despite the inactive zymogens of MMP-2 and MMP-9 existing as proforms due to the presence of latent propeptide domains, differences in prodomain cleavage and proteolytic activation by other MMPs or proteinases are acknowledged. Pro-MMP-2 activation can be mediated via a broad range of MMPs, most significantly through complex formation with membrane-type MMPs (MT-MMPs) and tissue inhibitor of metalloproteinases (TIMPs) [43, 44]. However, unlike pro-MMP-2, MT-MMP-/TIMP-dependent mechanisms of activation have not been described for pro-MMP-9, which is mediated by alternative MMP-driven mechanisms. Indeed, although MMP-2 is a key contributor to the proteolytic activation of pro-MMP-9, it is established that pro-MMP-9 predominantly remains in its latent proform during the culture of many different cell types, even in the presence of active MMP-2 [52]. Thus, a paradox remains as to the lack of active MMP-9 under cell culture conditions, although structural and catalytic differences between these gelatinases and their sequestration within the ECM have been suggested to contribute to this phenomenon [43, 44]. Nonetheless, the nonsenescent high proliferative/multipotent DPSC subpopulation, A3 (30 PDs), was unique in demonstrating pro-MMP-9 detection. Thus, as pro-MMP-9 is recognised to possess significant catalytic activity, even with the propeptide domain intact [53], the absence of active MMP-9 should not curtail the potential significance of pro-MMP-9 in facilitating type I collagen gel remodelling by these DPSC subpopulations. Indeed, this mechanism could be important for MSC maintenance in a quiescent state within the stem cell niche or in enhancing the 3D biomaterial scaffold remodelling/degradation capabilities of this DPSC subpopulation [15, 49]. Furthermore, pro-MMP-9 detection in the high proliferative/multipotent subpopulation, A3, at nonsenescent stages in its proliferative lifespan (at 30 PDs), may indicate another feature of the heterogeneous nature between DPSCs isolated from dental pulp tissues [4, 5, 17, 18, 21, 22]. However, we can only speculate at present as to the reasons underlying such differences in type I collagen gel contraction and MMP remodelling capabilities between high proliferative/multipotent and low proliferative/unipotent DPSCs, as patient donor features, developmental origins, stem cell niche sources, and/or the stages of commitment of these DPSC subpopulations would undoubtedly be potential influences on these cellular characteristics.

## 5. Conclusions

As with previous studies highlighting the phenotypic and genotypic heterogeneity which exists between high proliferative/multipotent and low proliferative/unipotent DPSC subpopulations utilising 2D monolayer cultures, this study demonstrates that heterogeneity is also evident in terms of the gel contraction capabilities and MMP expression/activity profiles of individual DPSC subpopulations, following seeding within 3D type I collagen gels. Despite distinct differences in proliferative capabilities and susceptibilities to replicative senescence between DPSC subpopulations previously being established during the extended 2D monolayer culture, contrasting proliferative responses were unexpectedly less apparent within 3D collagen gels. Such variations in type I collagen remodelling mechanisms by MMP-2 and MMP-9 between high proliferative/multipotent and low proliferative/unipotent DPSC subpopulations may reflect novel differences in their respective abilities to degrade biomaterial scaffolds and regulate cellular functions in 3D environments following *in vivo* transplantation, thereby influencing their individual regenerative properties overall. Consequently, these findings also aid our understanding of the molecular and phenotypic properties associated with high proliferative/multipotent DPSCs, which may be exploited for their selective screening and isolation from dental pulp tissues for regenerative medicine applications.

## Data Availability

The data used to support the findings of this study are available from the corresponding author upon request.
